# An Observational Retrospective Cohort Trial on 4,828 IVF Cycles Evaluating Different Low Prognosis Patients Following the POSEIDON Criteria

**DOI:** 10.3389/fendo.2019.00282

**Published:** 2019-05-08

**Authors:** Paolo Emanuele Levi-Setti, Irene Zerbetto, Annamaria Baggiani, Elena Zannoni, Laura Sacchi, Antonella Smeraldi, Emanuela Morenghi, Raffaella De Cesare, Alessandra Drovanti, Daniele Santi

**Affiliations:** ^1^Division of Gynaecology and Reproductive Medicine, Department of Gynaecology, Humanitas Fertility Center, Humanitas Research Hospital, Milan, Italy; ^2^Department of Obstetrics, Gynaecology and Reproductive Sciences, School of Medicine, Yale University, New Haven, CT, United States; ^3^Biostatistics Unit, Humanitas Research Hospital, Milan, Italy; ^4^Department of Biomedical, Metabolic and Neural Sciences, University of Modena and Reggio Emilia, Modena, Italy

**Keywords:** FSH, LH, ICSI, suboptimal responders, POSEIDON

## Abstract

**Objective:** To study the actual controlled ovarian stimulation (COS) management in women with suboptimal response, comparing clinical outcomes to the gonadotropins consume, considering potential role of luteinizing hormone (LH) addition to follicle-stimulating hormone (FSH).

**Design:** Monocentric, observational, retrospective, real-world, clinical trial on fresh intra-cytoplasmic sperm injection (ICSI) cycles retrieving from 1 to 9 oocytes, performed at Humanitas Fertility Center from January 1st, 2012 to December 31st, 2015.

**Methods:** COS protocols provided gonadotropin releasing-hormone (GnRH) agonist long, flare-up, short and antagonist. Both recombinant and urinary FSH were used for COS and LH was added according to the clinical practice. ICSI outcomes considered were: gonadotropins dosages; total, mature, injected and frozen oocytes; cumulative, transferred and frozen embryos; implantation rate; pregnancy, delivery and miscarriage rates. Outcomes were compared according to the gonadotropin regimen used during COS.

**Results:** Our cohort showed 20.8% of low responders, defined as 1–3 oocytes retrieved and 79.2% of “suboptimal” responders, defined as 4–9 oocytes retrieved. According to recent POSEIDON stratification, cycles were divided in group 1 (6.9%), 2 (19.8%), 3 (11.7%), and 4 (61.5%). The cohort was divided in 3 groups, according to the gonadotropin's regimen. Women treated with FSH plus LH showed worst prognostic factors, in terms of age, basal FSH, AMH, and AFC. This difference was evident in suboptimal responders, whereas only AMH and AFC were different among treatment groups in low responders. Although a different result, in terms of oocytes and embryos detected, major ICSI outcomes (i.e., pregnancy and delivery rates) were similar among groups of COS treatment. Outcomes were significantly different among Poseidon groups. Implantation, pregnancy and delivery rates were significantly higher in Poseidon group 1 and progressively declined in other POSEIDON groups, reaching the worst percentage in group 4.

**Conclusions:** In clinical practice, women with worst prognosis factors are generally treated with a combination of LH and FSH. Despite low prognosis women showed a reduced number of oocytes retrieved, the final ICSI outcome, in terms of pregnancy, is similarly among treatment group. This result suggests that the LH addition to FSH during COS could improve the quality of oocytes retrieved, balancing those differences that are evident at baseline.

**Clinical Trial Registration:** www.ClinicalTrials.gov, identifier: NCT03290911

## Introduction

The number of couples seeking help in assisted reproductive technologies (ART) is progressively increasing and about 1.5 million cycles are currently performed every year (1). ART starts with a controlled ovarian stimulation (COS) phase, in which the ovary is exogenously stimulated with gonadotropins at producing the largest number of oocytes to be used in embryo development. During COS, follicle stimulating hormone (FSH), human menopausal gonadotropin (hMG) and luteinizing hormone (LH) are variably used. However, a significant inter- and intra-individual different response to COS is largely demonstrated so far and the research practice is focused on the way to optimize ovarian response. In this improving process, two main challenges remain to be clarified nowadays. First, how women who poorly respond to COS could be identified? The first poor responders definition dates back to 1983 (2), although only in 2011 the first realistic attempt to define poor responders have been made by the scientific community of the European society of human reproduction and embryology (ESHRE) (3). Using this definition, poor responder women show at least two of the following criteria: (i) advanced age (>40 years), (ii) previous poor ovarian response (<3 oocytes retrieved), (iii) abnormal reserve test, detected as antral follicle count (AFC) < 5–7 or anti-Mullerian hormone (AMH) < 0.5–1.1 ng/mL (3). However, this classification fails to define all patients who experience poor response to ovarian stimulation. Recently, a new classification of low ovarian response has been proposed. Four subgroups have been identified considering quantitative and qualitative parameters, such as age, antral follicle count (AFC), and anti-Müllerian hormone (AMH) and ovarian response to previous stimulation cycle was performed, defined as a number of retrieved oocytes lower than 9 (4). These criteria could assist the clinician in the women classification, although they are still not useful to define the best COS treatment.

The second main challenge in ART is the appropriate gonadotropins stimulation needs to be used (5, 6). All COS protocols provide the exogenous FSH administration. Is FSH alone enough to induce multiple follicle development or a gonadotropins combination could improve the final outcome? Although wide consensus is reached about the tailored protocol, there is currently a lack of consensus on what represents the gold standard gonadotropins combination to COS. Indeed, COS schemes remain mainly empirical and a personalized medicine is still advocated in this setting (7). Best practices suggest the use 150–300 IU of gonadotropins daily, but no more than 450 IU daily, for a total of 9–10 days (8). These doses are generally applied considering the women expected response, using an average dosage of 150 IU for those younger and higher doses in women who are older or who are expected to have a poorer response. However, such approaches show poor clinical results and the ideal COS protocol is still under debate.

In the literature, a wide number of clinical trials evaluated the efficacy of gonadotropin combinations in COS. However, strong evidence in favor or contrast to gonadotropins combination is not reached so far (9). The analysis of large databases of infertile population could be useful to update the specification of the “best clinical practice” in ART. With this in mind, considering that large population based cohorts demonstrated that an oocyte yield of 10–15 oocytes in all age groups resulted in the most optimal live birth rate in fresh cycles (10), we decided to focus on women with low ovarian response according to recent diagnostic stratification (i.e., 9 oocytes retrieved). Thus, a real-world trial based on a large database is designed to collect data from the standard clinical practice in ART center in which all gonadotropins preparations were used alone or in combination. The main aim of this study is to analyse the actual COS management in women with not optimal response, comparing clinical outcomes to the gonadotropins consume.

## Materials and Methods

### Study Design

A monocentric, observational, retrospective, real-world, clinical trial was performed evaluating all ART cycles performed at the Humanitas Fertility Center from January 1st, 2012 and December 31st, 2015. All fresh cycles of intra-cytoplasmic sperm injection (ICSI), retrieving 1–9 oocytes were enrolled. Overall, this study enrolled women with low ovarian response, according to Patient-Oriented Strategies Encompassing IndividualizeD Oocyte Number (POSEIDON) stratification system (4, 11–13). Only ICSI cycles were included in the analyses considering that only in this ART methodology information on oocytes quality have been recorded. Moreover, inclusion criteria provided an age ≤44 years and a body mass index (BMI) between 18 and 27 kg/m^2^. The following exclusion criteria were considered: (i) age > 44 years, (ii) number of oocytes retrieved > 10 or < 1 or cycles suspended before retrieval, (iii) abnormal uterine cavity, (iv) endometriosis III-IV stage or adenomyosis, (v) testicular sperms, and (vi) PGT (Preimplantation Genetic Testing). Only fresh cycles were considered and pregnancies from frozen cycles were excluded from the analysis.

The study was approved by the Independent Ethical Committee of the Humanitas Institutional Clinic (Milan, Italy) (Trial registration number: NCT03290911). Informed and written consent was obtained from each patient after full explanation of the purpose and nature of all procedures used.

### COS Protocol

The COS protocol provided the use of recombinant FSH (rFSH), hMG or rFSH + recombinant LH (rFSH + rLH). The gonadotropin starting dose was determined according to ovarian reserve parameters, such as AMH, AFC, and BMI.

COS was performed using four different protocols: GnRH agonist long protocol; GnRH agonist short protocol; GnRH antagonist protocol; Flare-up GnRH agonist protocol. Most of antagonist COS starts with the use of combined oral contraceptives pretreatment.

Long GnRH agonist protocol was based on the administration of daily leuprorelin (Enantone die, Takeda, Italy) or Triptorelin Depot (3.75 mg IM, Decapeptyl®, Ipsen, Milan, Italy) on day 21 of the previous luteal phase of the stimulation cycle. When pituitary desensitization was achieved (14 days after the initiation of GnRH agonist), as evidenced by the absence of ovarian follicles >5 mm and endometrial thickness < 5.4 mm on transvaginal ultrasound examination, gonadotropin stimulation was initiated. In short GnRH agonist protocol the agonist (Leuprolin 0.1 mg/day) is administered from day 21 of the previous cycled and induction from day 1 or 2 of the cycle (day 1 being the start of the menstrual bleed) reducing the agonist dose to 0.05 mg/day and continuing with stimulation until the day of HCG administration. In the GnRH antagonist protocol, the first day of women spontaneous menstrual cycle or a withdrawal bleeding after receiving a low dose oral contraceptive, gonadotropin stimulation was initiated and when the leading follicle reached 13–14 mm in mean diameter, and/or plasma E2 exceeded 400 pg/ml, an injection of 0.25 mg of GnRH antagonist (Cetrotide®, Merck Serono S.p.A, Rome, Italy; Orgalutran®, Organon, MSD-Italy) was administered SC daily until the day of ovulation trigger.

Finally, in the Flare-up GnRH protocol, daily agonist started on cycle day 1 of the cycle with triptorelin (0.1 mg/day) and gonadotropins was started according to ovarian reserve parameter on day 2 of the cycle. A starting variable dose of gonadotropin (hMG (Meropur®, Ferring, Milan, Italy) or rFSH (Puregon®, MSD-Italy; Gonal-F, Merck Serono S.p.A., Rome, Italy) with or without the addition of r-LH for the first 4 days and then an individualized dose was administered according to the parameters resulting from transvaginal ultrasound and estradiol and progesterone levels until the day of ovulation trigger. The protocol of induction and the dose of gonadotropins administered were tailored on an individual basis according to patient's age, serum hormonal levels, and AFC. Transvaginal ultrasonography, estradiol and progesterone determinations were performed during COS. When at least three follicles with a mean diameter >18 mm were observed, 250 mcg of recombinant hCG (Ovitrelle; Merck Serono S.p.A.) was administered subcutaneously. Oocyte retrieval was performed transvaginal 36 h after hCG injection. Embryo transfer was performed day 3–day 5 after oocyte collection. Luteal phase was supported in all patients with vaginal progesterone (Crinone 8%; Merck Serono S.p.A. or Prometrium; Rottapharm). Serum hCG was assessed 2 weeks after embryo transfer and then every 48 h until a value over 1,000 mIU was detected and a vaginal ultrasound was scheduled 4 weeks after the embryo transfer.

### Parameters Detected

All anamnestic information was collected, with attention to the years of infertility and the indication to ART. Baseline women characteristics were collected after the last menstrual cycle before ART, such as female age, AFC, FSH, LH, estradiol, TSH, AMH and inhibin B serum levels. AMH and AFC were evaluated considering previous statements (14). The ART protocol applied was registered, considering the GnRH analog used, the FSH and LH doses used and the duration of the COS protocol. Finally, considering the ART outcomes, the following outcomes were considered: oocytes retrieved, oocyte nuclear maturity stage, injected, frozen and fertilized oocytes, transferred and frozen embryos. Implantation, pregnancy, live birth, and miscarriage / ectopic rates were finally collected. The implantation rate was calculated as the ratio between the number of gestational sacs identified at this time and the number of embryos transferred. Clinical pregnancy was defined a pregnancy as visualization of one or more gestational sacs or definitive clinical signs of pregnancy. It includes ectopic pregnancy as defined by The International Committee for Monitoring Assisted Reproductive Technology (ICMART) and the World Health Organization (WHO) Revised, Glossary on ART Terminology, 2009 [21]. Miscarriage rate and ectopic pregnancies, per clinical pregnancy, were defined as the proportion of patients who failed to continue development before 20 weeks of gestation in all clinical pregnancies. Live birth was defined as the delivery of a fetus with signs of life after 20 completed weeks of gestational age.

### Statistical Analysis

The entire dataset was first evaluated to select each single couple treated with ART. These couples represented the entire cohort of patients evaluated by the study.

Descriptive analyses were performed considering the entire cohort of patients. Continuous variables distribution was evaluated with Kolmogorov-Smirnov test. According to the not normal distribution, continuous variables were compared with Kruskal Wallis non-parametric test. *Post-hoc* analyses were performed by Tukey test. Continuous variables are expressed as mean ± standard deviation. Categorical variables were compared using Fisher exact test and they were expressed as number (percentage). Multiple linear stepwise analyses were performed considering implantation rate as dependent variable and other parameters as independent variables. These analyses were repeated, considering the following POSEIDON groups: (1) age <35 years with adequate ovarian reserve (AFC>5 and AMH>1.2 ng/mL) and 9 oocytes retrieved; (2) age >35 years with adequate ovarian reserve (AFC>5 and AMH>1.2 ng/mL) and 9 oocytes retrieved; (3) age <35 years with poor ovarian reserve (AFC <5 and AMH < 1.2 ng/mL); (2) age >35 years with poor ovarian reserve (AFC <5 and AMH < 1.2 ng/mL).

Statistical analysis was performed using the “Statistical Package for the Social Sciences” software for Macintosh (version 21.0; SPSS Inc., Chicago, IL). Considering that multiple hypotheses were tested together, and multiple analyses were performed, the statistical significance was evaluated after correction using Bonferroni test. Thus, nine endpoints were consecutively evaluated, and the *p*-values was considered statistically significant when *p* < 0.0005. Moreover, considering the real-world data registration considers both repeated (i.e., multiple cycles performed for the same couples) and missing data, the missing not at random (MNAR) approach was used to adjust analyses (15). To this purpose, the Expectation-Maximization method was applied, creating a new dataset in which all missing values are estimated by the maximum likelihood methods (16).

## Results

**Figure 1:**
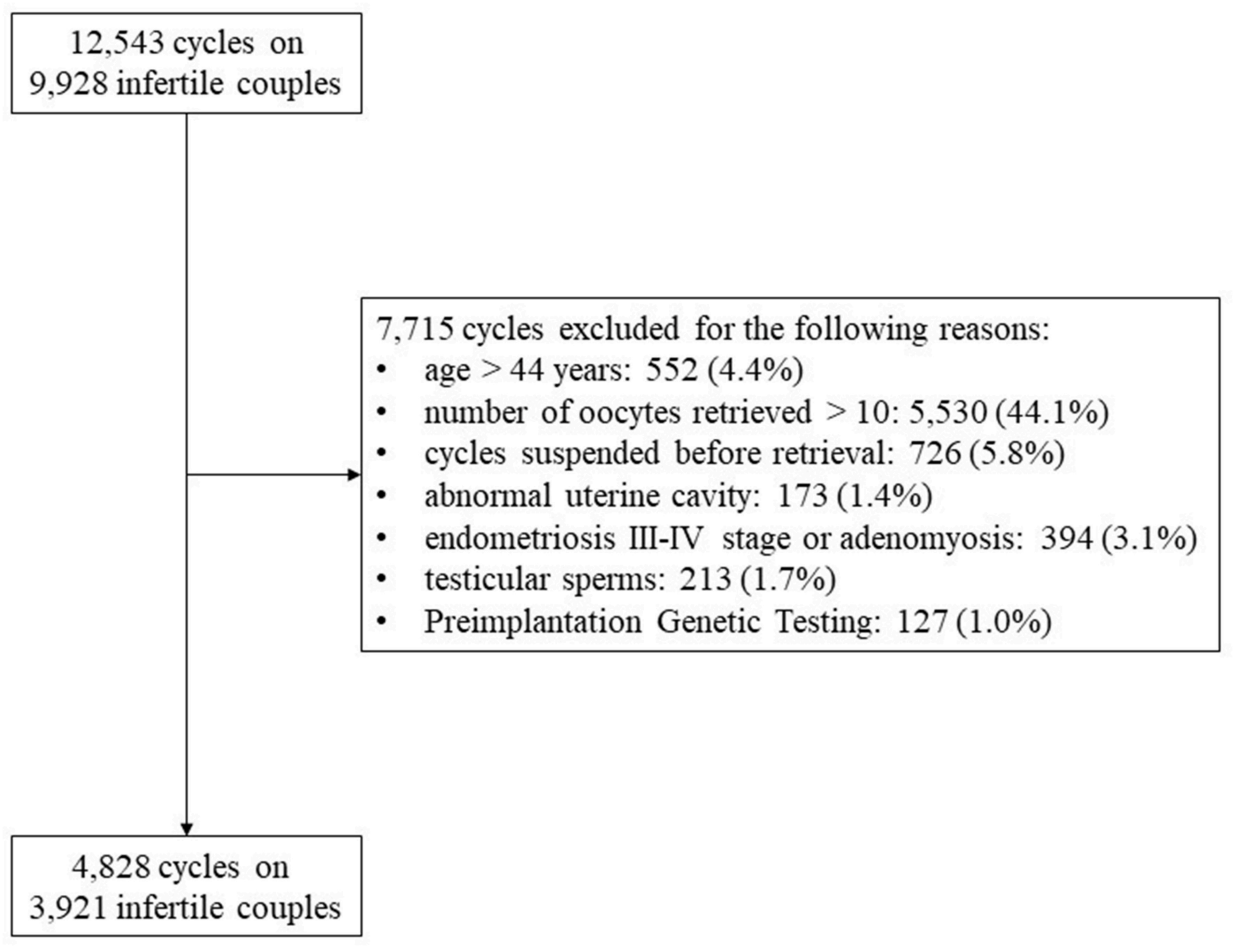
Study flow chart. ^*^p<0.05.

Twelve thousand five hundred and forty-three ART cycles were performed from January 1st, 2012 and December 31st, 2015 on 9,928 infertile couples. Finally, 4,828 cycles fulfilled inclusion and exclusion criteria and represented the final cohort evaluated. Figure 1 showed the study flow chart and reported the reasons for exclusion (Figure 1).

The mean infertility duration was 4.61 + 7.18 years, with a mean age of 37.67 + 3.68 years for the female and 40.02 + 5.37 years for the male partner. The mean number oocytes retrieved was 5.55 + 2.40 (from a minimum 1 to a maximum of 9). The transfer was suspended in 528 cycles (10.9%), due to sperms absence in 15 cycles (0.3%), oocytes low quality in 131 (2.7%), no fertilization in 241 (5.0%), lack of oocyte cleavage in 57 (1.2%), embryos not developed in 13 (0.3%), OHSS risk in 51 (1.0%), and complications in 20 (0.4%). Severe OHSS was developed in 4 cycles (0.1%).

The entire cohort showed the 20.8% of low responders (1,006 women who retrieved 1–3 oocytes) and the 79.2% of women with a “suboptimal” response (3,822 patients who collected from 4 to 9 oocytes). The cohort of ART cycles was divided in the following 3 groups, according to the gonadotropins regimen: (i) group 1: FSH alone (3,338 cycles, 69.1%); (ii) group 2: hMG alone (678 cycles, 14.0%); (iii) group 3 FSH combined to LH (812 cycles, 16.8%). Moreover, according to recent patients stratification, the 18.6% (897 women) of women enrolled were below than 35 years old and the 73.3% (3,538 women) showed low ovarian reserve parameters, in terms of AFC and AMH. Thus, considering the POSEIDON stratification, group 1 included 233 patients (6.9%), group 2 958 (19.8%), group 3 565 (11.7%) and group 4 2,973 (61.5%).

### Comparison Among Gonadotropins Groups

**Figure 2:**
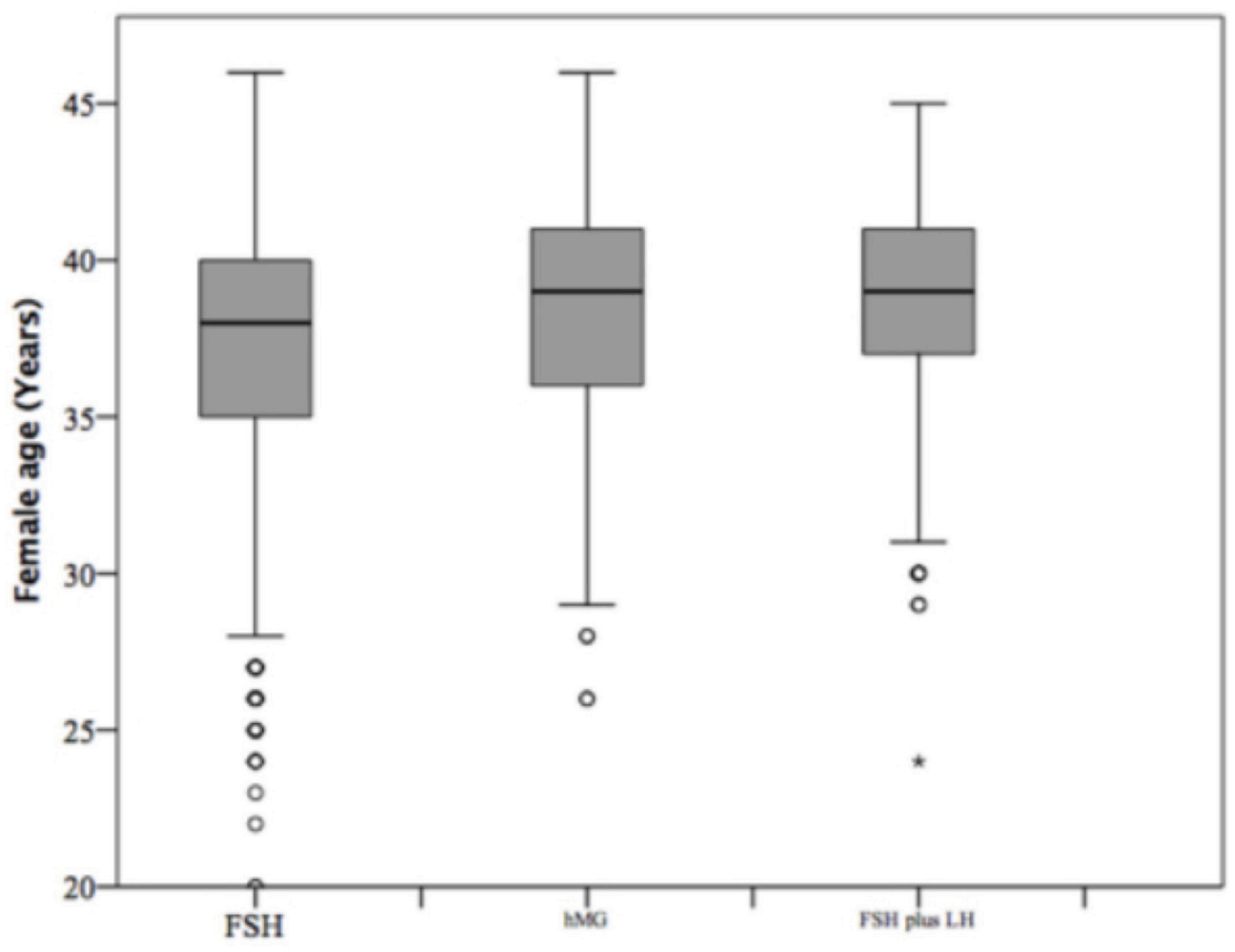
Female age among groups of patients, divided according to gonadotropins regimen chosen.

**Figure 3:**
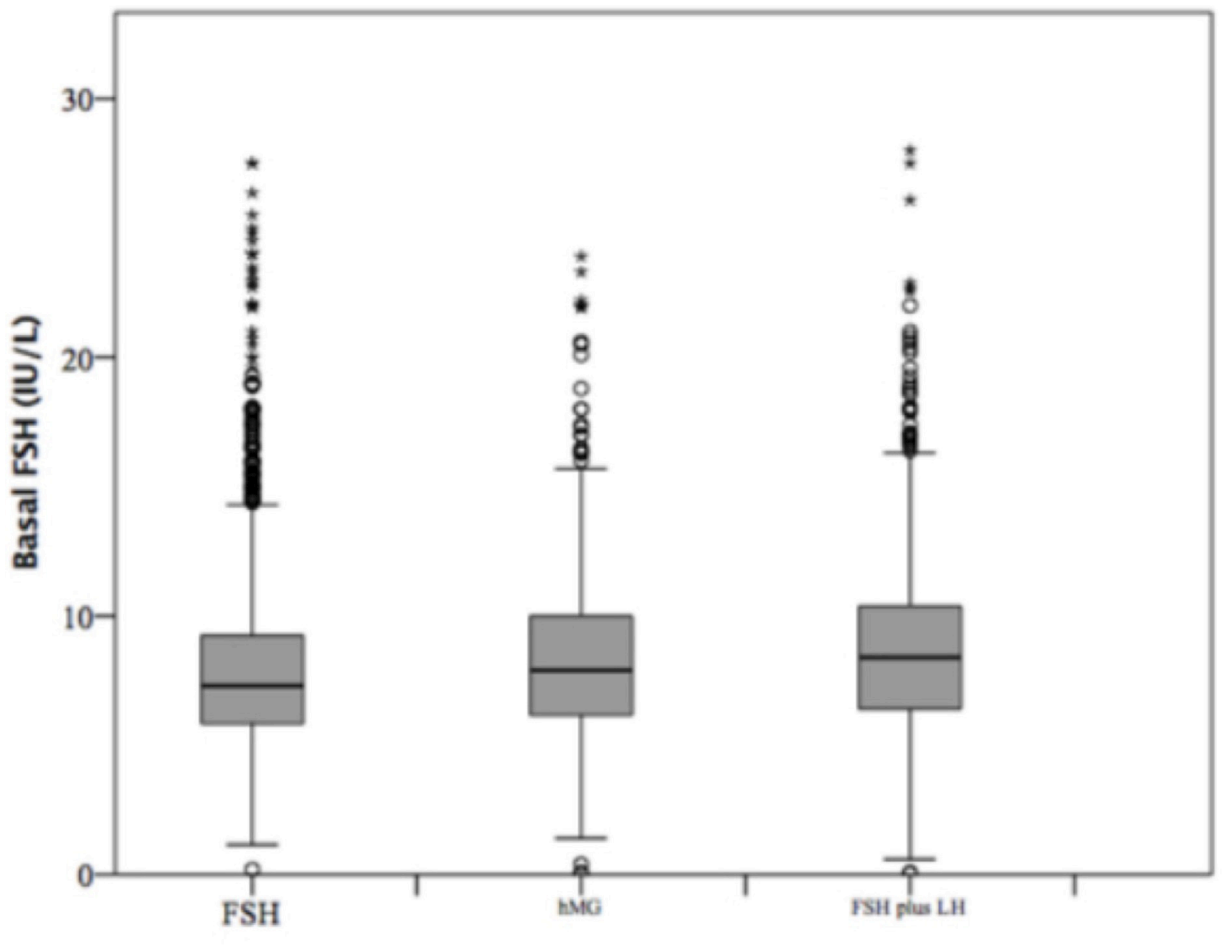
Basal follicle stimulating hormone (FSH) serum levels among groups of patients, divided according to gonadotropins regimen chosen.

**Figure 4:**
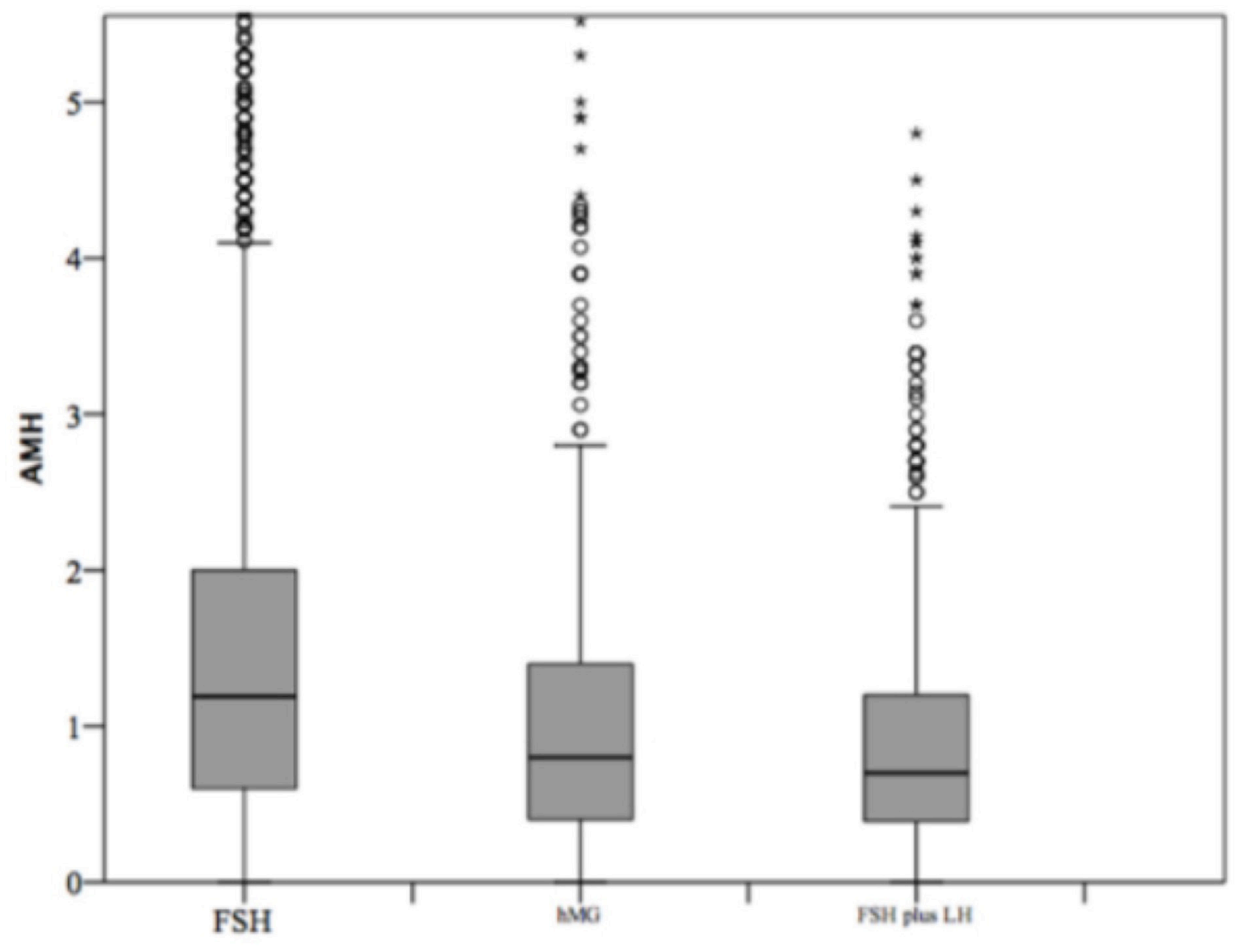
Anti-Mullerian hormone (AMH) among groups of patients, divided according to gonadotropins regimen chosen. *p<0.05.

**Figure 5:**
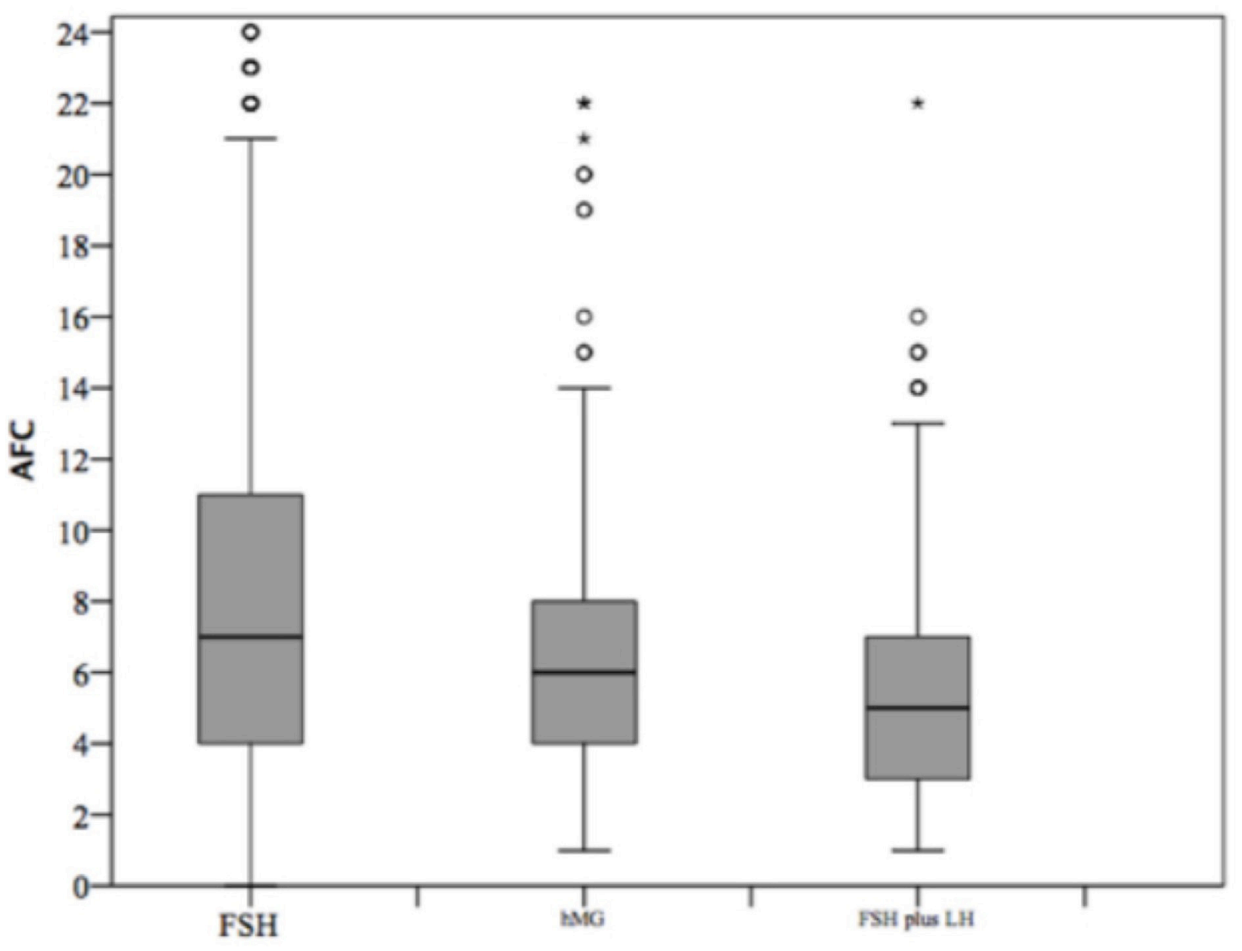
Antral follicle count (AFC) among groups of patients, divided according to gonadotropins regimen chosen. *p<0.05.

At baseline, a significant difference among groups was detected. Female age was significantly higher in group 3 compared to group 1 and 2 (*p* < 0.001 and *p* < 0.001, respectively) (Table 1). Moreover, female age was significantly higher in hMG group compared to FSH group (*p* < 0.001; Figure 2). Basal FSH serum levels were significantly higher in group 3, compared to groups 1 and 2 (*p* < 0.001 and *p* < 0.001, respectively) and lower levels were detected in group 1 compared to 2 (*p* = 0.001; Figure 3). Similar trend was detected for AMH and AFC, which were significantly higher in group 1 and progressively decreased until group 3 (Figures 4, 5). On the contrary, basal LH did not change among groups (Table 1). These results, taken together, suggest that a different clinical approach is generally applied, preferring the LH addition to FSH when a low response is expected.

**Table 1:** Patients and ART outcomes.

**Variable**	**FSH alone**	**hMG**	**FSH+LH**	***p*-value**
Number of cycles n (%)	3,338 (69.1%)	678 (14.0%)	812 (16.9%)	-
Female Age (years)	37.26 ± 3.77	38.49 ± 3.47	38.70 ± 3.12	**<0.001**
Male Age (years)	39.69 ± 5.40	40.76 ± 5.53	40.74 ± 4.99	**<0.001**
Basal FSH (IU/L)	7.88 ± 3.16	8.44 ± 3.51	8.91 ± 3.66	**<0.001**
Basal LH (IU/L)	5.80 ± 7.65	5.19 ± 4.70	5.70 ± 4.81	0.641
AMH (ng/mL)	1.62 ± 1.60	1.11 ± 1.16	0.98 ± 1.06	**<0.001**
AFC	8.31 ± 5.99	6.45 ± 3.82	5.33 ± 3.09	**<0.001**
Number of ART cycles n (%)	2.47 ± 1.13	2.32 ± 1.07	1.98 ± 1.02	0.091
Induction protocol n (%)				**<0.001**
*Antagonist cycle*	1,617 (48.4%)	323 (47.6%)	307 (37.8%)	
*Agonist flare-up*	750 (22.5%)	198 (29.2%)	258 (31.8%)	
*Agonist long*	409 (12.3%)	56 (8.3%)	86 (10.6%)	
*Agonist short*	38 (1.2%)	9 (1.3%)	30 (3.7%)	
Induction length (days)	11.57 ± 2.92	11.47 ± 1.87	12.01 ± 3.97	0.123
Total FSH doses (IU)	3,319 ± 1,351	3,573 ± 1,240	3,217 ± 1,033	**<0.001**
Total LH dose (IU)	0	0	1423 ± 562	**<0.001**
FSH dose/oocytes ratio	798.88 ± 824.60	935.37 ± 786.48	845.61 ± 795.86	**<0.001**
Retrieved oocytes	5.78 ± 2.45	5.13 ± 2.17	4.96 ± 2.11	**<0.001**
MII oocytes	4.32 ± 2.10	3.87 ± 1.91	3.77 ± 1.86	**<0.001**
MII oocytes/oocytes retrieved	0.75 ± 0.24	0.77 ± 0.24	0.77 ± 0.24	0.042
Injected oocytes	4.21 ± 2.09	3.82 ± 1.92	3.69 ± 1.86	**<0.001**
Frozen oocytes	0.04 ± 0.46	0.03 ± 0.36	0.03 ± 0.40	0.816
Fertilized oocytes	3.03 ± 1.89	2.73 ± 1.72	2.60 ± 1.65	**<0.001**
Cumulative embryos	2.15 ± 1.17	2.06 ± 1.11	2.03 ± 1.09	0.009
Transferred embryos	1.84 ± 0.94	1.85 ± 0.96	1.87 ± 0.99	0.785
Frozen embryos	0.32 ± 0.77	0.22 ± 0.61	0.17 ± 0.53	0.054
Implantation rate %	13 ± 28	12 ± 26	11 ± 26	0.252
Biochemical pregnancies n (%)	663 (19.9)	126 (18.6%)	145 (17.9%)	0.353
Deliveries n (%)	477 (14.3%)	90 (13.3%)	102 (12.6%)	0.926
Miscarriages n (%) ^*^	174 (5.2%)	33 (4.9%)	41 (5.0%)	0.954
Miscarriages n (%) ^**^	174 (26.2%)	33 (26.2%)	41 (28.3%)	
Ectopic pregnancies n(%)^*^	11 (0.3%)	3 (0.4%)	2 (0.2%)	0.960
Ectopic pregnancies n(%)^**^	11 (1.7%)	3 (2.4%)	2 (1.4%)	

Data are expressed as mean ± standard deviation or number (percentage).

* % Considering the total number of cycles.

** % Considering the number of clinical pregnancies.

*Bold values, represent statistical significant p-values*.

The GnRH analog used to induce COS was different among groups. The GnRH antagonist was generally chosen when FSH or hMG were used. On the contrary, when the FSH and LH combination was selected, agonist flare-up and antagonist protocols were equally used (Table 1). The duration of gonadotropins administration was similar among groups (*p* = 0.123; Table 1), whereas an obvious difference in gonadotropin dosages was detected. FSH doses were significantly higher in group 2 compared to 1 and 3 (*p* < 0.001 and *p* < 0.001, respectively), whereas no FSH dosages differences were seen between groups 1 and 3 (*p* = 0.107). This difference translated in a significant reduction in the FSH dose needed for each oocyte retrieved (Table 1), comparing group 3 with groups 2 (*p* < 0.001).

**Figure 6:**
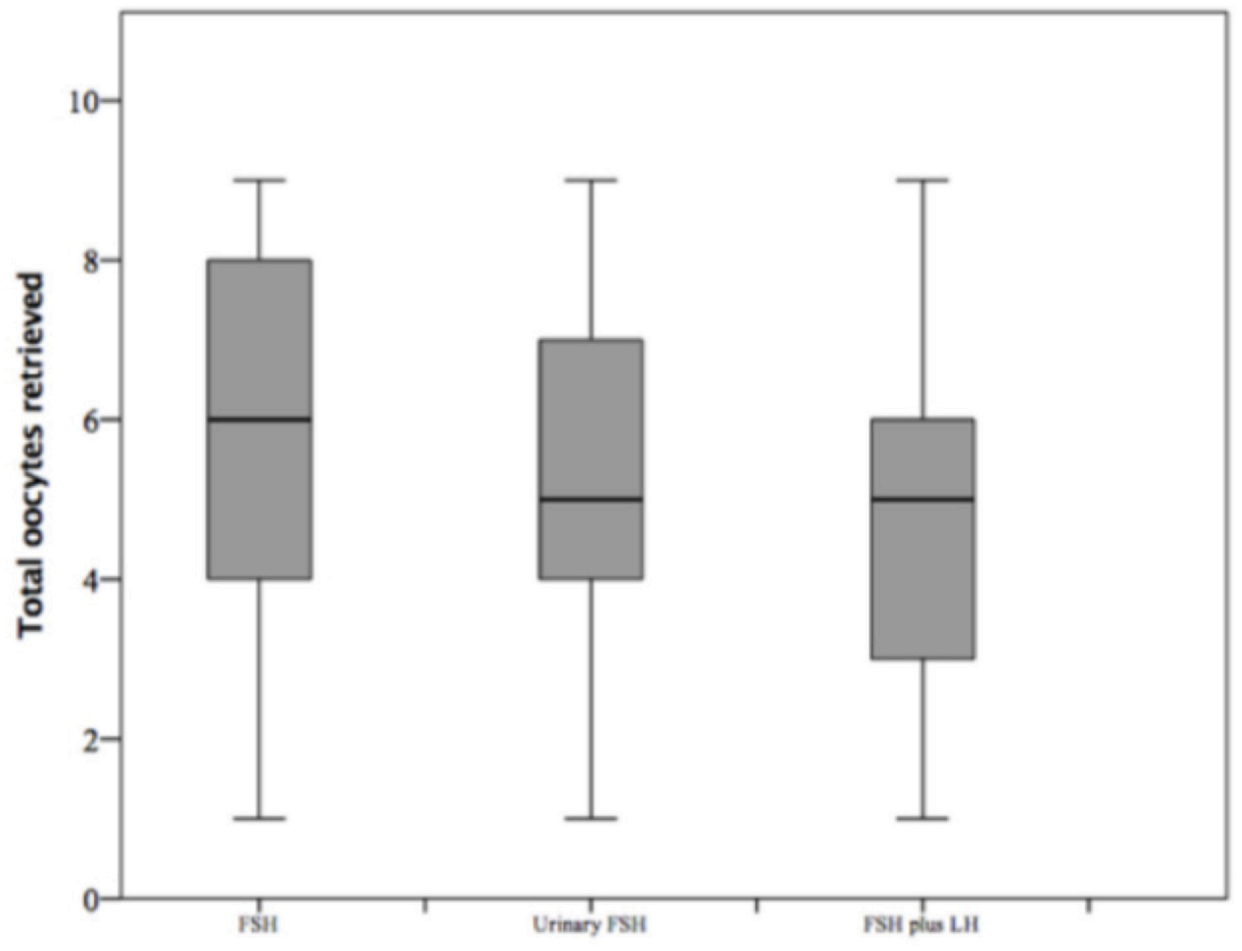
Retrieved oocytes among groups of patients, divided according to gonadotropins regimen chosen.

The number of oocytes retrieved was higher in group 1 compared to group 2 and 3 (*p* < 0.001 and *p* < 0.001, respectively) (Table 1 and Figure 6). However, no significant differences were seen between groups 2 and 3 (*p* = 0.319), suggesting that despite the baseline differences between these two groups, the addition of LH to FSH retrieved a relative higher oocytes number compared to hMG. Similarly, the number of MII oocytes reflected the total oocytes number, with a higher retrievement in group 1 compared to groups 2 and 3 (*p* < 0.001 and *p* < 0.001) and a similar number between groups 2 and 3 (*p* = 0.620). Interestingly, the MII oocytes on total oocytes retrieved ratio was not different among groups (Table 1). Again, similar trend was observed for injected and fertilized oocytes (Table 1). Despite the differences between group 1 and other groups, the number of cumulative, transferred and frozen embryos did not differ among groups (Table 1), suggesting that the addition of LH improve the oocytes quality and capability to develop embryos. This hypothesis was further confirmed by the similar results among groups obtained for implantation, pregnancy and delivery rates (Table 1).

### Comparison Among Patients Stratification

Considering only women with low response (1–3 oocytes retrieved), despite the different baseline characteristics, all ART outcomes evaluated did not differ among groups (Table 2). On the contrary, considering women with a “suboptimal” response (oocytes retrieved between 4 and 9), the three groups showed significant differences (Table 3). First, baseline characteristics (i.e., age, FSH, AMH and AFC) were significantly impaired in group 3 compared to group 1 and 2 and in group 2 compared to group 1. Considering the ART outcomes, the number of total, MII, injected and fertilized oocytes was significant higher when FSH was used alone (group 1), compared to hMG (group 2) or FSH plus LH (group 3), while no differences were seen between groups 2 and 3. Moreover, other endpoints (i.e., embryos, implantation, pregnancy and delivery rates) were not different among groups (Table 3). These results suggest that the LH addition to FSH reduce the baseline differences among women obtaining similar outcomes to those women with better prognosis. These differences were maintained after analysis statistical adjustment for women age, AFC, AMH and GnRH protocols used. Finally, multivariate analyses were not able to identified independent variables able to modifying independent one.

**Table 2:** ART outcomes in **low** responder women.

**Variable**	**FSH**	**hMG**	**FSH+LH**	***p*-value**
Number of cycles n (%)	623 (61.9%)	164 (16.3%)	219 (21.8%)	
Female Age (years)	38.00 ± 3.45	38.74 ± 3.35	38.74 ± 2.96	0.002
Male Age (years)	40.01 ± 5.38	41.10 ± 6.45	40.36 ± 4.77	0.070
Basal FSH (IU/L)	9.01 ± 3.97	9.61 ± 3.99	9.57 ± 3.91	0.083
Basal LH (IU/L)	5.36 ± 2.75	5.09 ± 2.05	6.11 ± 2.47	0.019
AMH (ng/mL)	± 1.16	0.69 ± 0.76	0.66 ± 0.82	** < 0.001**
AFC	6.29 ± 4.68	5.58 ± 3.52	4.80 ± 2.44	** < 0.001**
Total FSH doses (IU/L)	3,893 ± 1,375	3,621 ± 1,289	3,269 ± 1,046	** < 0.001**
Total LH dose (IU/L)	0	0	1,484 ± 575	** < 0.001**
FSH dose/oocytes ratio	1,944 ± 1,276	1,847 ± 1,069	1,555 ± 825	** < 0.001**
Oocytes retrieved	2.36 ± 0.73	2.27 ± 0.74	2.37 ± 0.69	0.327
MII oocytes	1.78 ± 0.91	1.84 ± 0.85	1.86 ± 0.86	0.481
MII oocytes/oocytes retrieved	0.75 ± 0.32	0.80 ± 0.29	0.78 ± 0.30	0.099
Injected oocytes	1.76 ± 0.94	1.81 ± 0.88	1.84 ± 0.87	0.488
Frozen oocytes	0.01 ± 0.17	0.02 ± 0.17	0	0.495
Fertilized oocytes	1.31 ± 0.94	1.20 ± 0.90	1.41 ± 0.89	0.075
Cumulative embryos	1.25 ± 0.91	1.16 ± 0.88	1.34 ± 0.86	0.149
Transferred embryos	1.22 ± 0.90	1.212 ± 0.87	1.32 ± 0.87	0.084
Frozen embryos	0.03 ± 0.22	0.04 ± 0.23	0.01 ± 0.12	0.418
Implantation rate	10.05 ± 27.00	9.38 ± 25.08	13.48 ± 29.68	0.305
Biochemical pregnancies n (%)	65 (10.4%)	17 (10.4%)	35 (16.0%)	0.186
Deliveries n (%)	40 (6.4%)	11 (6.7%)	21 (9.6%)	0.948
Miscarriage n (%) ^*^	24 (3.8%)	6 (3.7%)	14 (6.4%)	0.921
Miscarriage n (%)^**^	24 (36.9%)	6 (35.3%)	14 (40.0%)	
Ectopic pregnancies n (%)^*^	1 (0.2%)	0 (0%)	0 (0%)	0.978
Ectopic pregnancies n (%)^**^	1 (1.5%)	0 (0%)	0 (0%)	

Data are expressed as mean ± standard deviation or number (percentage).

* % Considering the total number of cycles.

** % Considering the number of clinical pregnancies.

*Bold values, represent statistical significant p-values*.

**Table 3:** ART outcomes in “suboptimal” responder women.

**Variable**	**FSH**	**hMG**	**FSH+LH**	***p*-value**
Number of cycles n (%)	2,715 (71.0%)	514 (13.4%)	593 (15.5%)	
Female Age (years)	37.09 ± 3.84	38.41 ± 3.50	38.68 ± 3.18	**<0.001**
Male Age (years)	39.62 ± 5.40	40.65 ± 5.20	40.88 ± 45.07	**<0.001**
Basal FSH (IU/L)	7.62 ± 2.88	8.07 ± 3.26	8.67 ± 3.53	**<0.001**
Basal LH (IU/L)	5.90 ± 9.53	5.22 ± 5.24	5.55 ± 4.04	0.674
AMH (ng/mL)	1.77 ± 1.66	1.25 ± 1.23	1.10 ± 1.12	**<0.001**
AFC	8.74 ± 6.15	6.73 ± 3.88	5.84 ± 3.28	**<0.001**
Total FSH doses (IU/L)	3,187 ± 1,312	3,559 ± 1,225	3,197 ± 1,028	**<0.001**
Total LH dose (IU/L)	0	0	1,401 ± 556	**<0.001**
FSH dose/oocytes ratio	536 ± 303	644 ± 319	583 ± 249	**<0.001**
Oocytes retrieved	6.56 ± 1.67	6.04 ± 1.62	5.92 ± 1.59	**<0.001**
MII oocytes	4.90 ± 1.85	4.51 ± 1.69	4.47 ± 1.63	**<0.001**
MII oocytes/oocytes retrieved	0.75 ± 0.21	0.75 ± 0.22	0.76 ± 0.21	0.314
Injected oocytes	4.77 ± 1.86	4.46 ± 1.71	4.37 ± 1.65	**<0.001**
Frozen oocytes	0.05 ± 0.50	0.04 ± 0.40	0.05 ± 0.47	0.844
Fertilized oocytes	3.42 ± 1.83	3.22 ± 1.63	3.04 ± 1.65	**<0.001**
Cumulative embryos	2.36 ± 1.12	2.35 ± 1.02	2.29 ± 1.06	0.316
Transferred embryos	1.99 ± 0.89	2.09 ± 0.87	2.07 ± 0.95	0.017
Frozen embryos	0.38 ± 0.83	0.27 ± 0.67	0.22 ± 0.60	0.072
Implantation rate	13.89 ± 27.69	12.97 ± 26.70	10.86 ± 24.33	0.060
Biochemical pregnancies n (%)	598 (22.0%)	109 (21.2%)	110 (18.5%)	0.170
Deliveries n (%)	437 (16.1%)	79 (15.4%)	81 (13.7%)	0.981
Miscarriage n (%)^*^	150 (5.5%)	27 (5.2%)	27 (4.5%)	0.962
Miscarriage n (%)^**^	150 (25.1%)	27 (24.8%)	27 (24.5%)	
Ectopic pregnancies n (%)^*^	10 (0.4%)	3 (0.6%)	2 (0.3%)	0.941
Ectopic pregnancies n (%)^**^	10 (1.7%)	3 (2.7%)	2 (1.8%)	

Data are expressed as mean ± standard deviation or number (percentage).

* % Considering the total number of cycles.

** % Considering the number of clinical pregnancies.

*Bold values, represent statistical significant p-values*.

All ART outcomes were significantly different among groups after patients stratification (Table 4). *Post-hoc* analyses showed better ART outcomes in group 1 compared to other three groups and group 4 showed lower ART outcomes compared to others. Accordingly, implantation, pregnancy and delivery rates were significantly higher in group 1 and progressively declined among POSEIDON groups, reaching the worst percentage in group 4 (Table 4). However, the COS stimulation in these groups was not homogeneous, showing higher FSH and lower LH doses in women belonged to group 1 (Table 4), confirming the previous suggestion that clinician usually adapted the gonadotropin stimulation to the women ovarian reserve.

**Table 4:** ART outcomes women divided according to POSEIDON stratification.

**Variable**	**Group 1**	**Group 2**	**Group 3**	**Group 4**	***p*-value**
Total FSH doses (IU/L)	2,182 ± 998	2,882 ± 1,127	3,282 ± 1,380	3,624 ± 1,329	**<0.001**
Total LH dose (IU/L)	50 ± 236	137 ± 406	219 ± 545	351 ± 654	**<0.001**
Oocytes retrieved	6.68 ± 1.91	6.38 ± 2.09	5.69 ± 2.22	5.13 ± 2.21	**<0.001**
FSH dose/oocytes ratio	380 ± 280	561 ± 520	779 ± 709	970 ± 874	**<0.001**
MII oocytes	5.11 ± 2.05	4.87 ± 2.02	4.21 ± 2.03	3.83 ± 1.97	**<0.001**
MII oocytes/oocytes retrieved	0.76 ± 0.22	0.76 ± 0.21	0.74 ± 0.23	0.75 ± 0.25	0.187
Injected oocytes	5.01 ± 2.01	4.74 ± 2.01	4.09 ± 2.02	3.74 ± 1.96	**<0.001**
Frozen oocytes	0.01 ± 0.17	0.07 ± 0.62	0.03 ± 0.39	0.03 ± 0.39	0.097
Fertilized oocytes	3.64 ± 1.92	3.46 ± 1.89	2.95 ± 1.89	2.65 ± 1.73	**<0.001**
Cumulative embryos	2.31 ± 1.14	2.36 ± 1.20	2.00 ± 1.15	2.05 ± 1.12	**<0.001**
Transferred embryos	1.69 ± 0.63	1.96 ± 0.99	1.59 ± 0.71	1.88 ± 0.99	**<0.001**
Frozen embryos	0.63 ± 1.00	0.40 ± 0.90	0.42 ± 0.85	0.17 ± 0.54	**<0.001**
Implantation rate	20.64 ± 35.17	13.03 ± 26.19	19.70 ± 26.19	10.57 ± 24.48	**<0.001**
Biochemical pregnancies n (%)	91 (30.1%)	199 (23.9%)	144 (29.1%)	500 (19.2%)	**<0.001**
Deliveries n (%)	82 (27.1%)	136 (16.3%)	119 (24.0%)	334 (12.8%)	**<0.001**
Miscarriage n (%)^*^	8 (2.6%)	59 (7.1%)	23 (4.6%)	158 (6.1%)	**<0.001**
Miscarriage n (%)^**^	8 (8.8%)	59 (29.6%)	23 (16.0%)	158 (31.6%)	
Ectopic pregnancies n (%)^*^	1 (0.3%)	4 (0.5%)	2 (0.4%)	9 (0.3%)	**<0.001**
Ectopic pregnancies n (%)^**^	1 (1.1%)	4 (2.0%)	2 (1.4%)	9 (1.8%)	

Data are expressed as mean ± standard deviation or number (percentage).

* % Considering the total number of cycles.

** % Considering the number of clinical pregnancies.

*Bold values, represent statistical significant p-values*.

## Discussion

Our study supports the substantial effect of LH addition to FSH during COS in a large real-world ART setting. Here, 4,828 ICSI cycles performed on women with less or equal than 9 oocytes retrieved have been fully evaluated. Despite these women are known to poorly respond to COS protocol, baseline clinical characteristics guide the clinician's decision about the best COS approach. Indeed, women with higher age, higher FSH basal levels, lower AMH serum levels and lower AFC (representing the 30.9% of the entire cohort) are generally treated with gonadotropin combination. In particular, the worst clinical baseline picture, occurring in 16.8% of the cohort, is preferentially treated with FSH plus LH, instead of hMG (i.e., FSH plus hCG). On the contrary, women with a best baseline clinical picture (the 69.1% of the entire cohort) are treated with FSH alone. This clinical approach is further confirmed stratifying patients considering more recent classification, dividing women according to the number oocytes retrieved and the ovarian reserve (i.e., AFC and AMH serum levels) (4). However, despite the unbalance between patients' baseline characteristics, the final ART outcomes are similar among the three COS approaches evaluated. Indeed, FSH alone, hMG or FSH plus LH reached the same result, in terms of embryos number, implantation, pregnancy and delivery rates. This is extremely important considering that the number of oocytes retrieved is an important prognostic variable for ART success (17, 18). The LH addition to FSH balances the final ART outcome among groups, despite the women clinical baseline differences. Interestingly, the use of hMG is usually chosen for those women with expected results within FSH alone or FSH-LH combination (14.0%). According, hMG obtains similar results to other groups, suggesting that the hCG-LH activity added to FSH during COS can improve the final ART outcome. However, the use of hMG shows a higher FSH on oocytes ratio, suggesting that this approach leads to a higher FSH consume to obtain stimulation like FSH alone or FSH plus LH.

The beneficial action of LH on COS is particularly evident dividing women enrolled in two clinical groups: low and suboptimal responders. Low responders should represent women with 1–3 oocytes retrieved, whereas suboptimal those with 4–9 oocytes. Indeed, Sunkara et al. established that women with 4–9 oocytes retrieved result in acceptable live birth rates, ranging from 15 to 36%, although the level of response to stimulation which is not ideal (18) Indeed, age-matched women with 10–15 oocytes retrieved obtain a live birth rates 20–30% higher (18). When suboptimal responders are considered, the beneficial LH action is confirmed. Indeed, in this subgroup, women are treated with FSH alone when the better outcome is expected, whereas the FSH-LH combination is preferred when an impaired baseline picture is evident, such as higher age, FSH basal levels and lower AMH and AFC. Despite these differences, the LH addition improves the ART outcome, obtaining similar results in terms of embryos number, implantation, pregnancy and delivery rates. On the other hand, low responders, although reduced of number (1,096 women), show a slight baseline difference among groups of COS approaches, only in terms of AMH and AFC. According to the reduced baseline difference, the final ART outcome remains similar among groups. However, no differences are observed also in terms of oocytes number and quality. Thus, it is probable that the LH addition improves the entire COS process, reducing the differences among women according to the baseline characteristics. In this context, a further evaluation of potential single nucleotide polymorphisms (SNP) effect on ART outcomes must be considered. Indeed, several trials suggested so far that gonadotropin effect during ART Could be modulated by SNP on gonadotropin and gonadotropin-receptor genes (19–25).

Nowadays in ART it is well known that COS schemes should be personalized to each woman to ensure the highest chance of live birth rate (7, 26). Several models have been developed with the aim at predicting outcomes for the infertile patient and at tailoring gonadotropins stimulation (27–29). These models involve similar, well-established predictors, such as female age, duration of infertility, number of previous successful or unsuccessful ART cycles, pregnancy history and whether infertility was caused by tubal pathology. However, they still need external validation and they are not routinely clinically applied (30, 31). These models are well designed on poor responders, although a well-standardized approach remains far to be completely elucidated (32, 33). However, no specific large datasets are available to build predictive models for poor responder patients. This is the first real-world evaluation of more than twelve thousand ART cycles. This paper is a perfect snapshot of population who typically attends ART centers: woman average age is higher than 37 years and the entire cohort showed the 38.5% of patients with a not-optimal response to ovarian stimulation ( ≤ 9 oocytes retrieved). Thus, alongside the wide number of women enrolled, our trial gives an interesting focus on the actual COS approach in clinical practice.

A large number of clinical trials and meta-analyses have been performed at comparing different gonadotropins combinations in terms of COS outcome (34–40). These publications focused on a wide range of heterogeneous studies, evaluating different endpoints, setting and patient characteristics. Recently, a more-comprehensive meta-analysis on FSH plus LH during COS has been performed, including 70 clinical trials and detecting a clear effect of LH on the final ART outcomes (41). Whether FSH alone obtains higher oocyte number, the FSH-LH combination leads to a higher pregnancy rate (41). Thus, the LH addition seems to increase the selective pressure on follicular selection exerted by the two gonadotropins together, improving oocyte quality. Here, we support these previous results in a real-world setting. Indeed, our results confirm in a clinical setting the LH molecular action widely demonstrated in the literature. Indeed, through the specific receptor binding, LH leads to highest activation of ERK1/2 and AKT-pathway and a final proliferative and anti-apoptotic signal (42, 43). LH exerts a proliferative action at molecular level, leading to a better ART outcome. This study shows three main limitations. First, this study was retrospectively designed, thus possible selection and performed biases should be considered. Indeed, clinical trials should provide an a priori study design, which help in the selection of patients, limiting the inter- and intra-individual differences. Second, the groups of treatment show a significant baseline differences, in terms of those parameters widely associated to the final ART outcomes. Thus, the groups are not completely comparable at baseline and consequently a clear advantage to one treatment to another is not clearly demonstrated. Third, the gonadotropin administration is not standardized in each group, but each woman was treated with a tailored therapy.

In conclusion, in our work, a deep and accurate description of ovarian response to COS in a large population of women undergoing ART is performed. Here, more than 4,000 cycles of women with sub-optimal response are detected, defined as those women who retrieve from 1 to 9 oocytes. Using this large database, for the first time a beneficial effect of LH addition to FSH during COS raises from the clinical practice. In particular, the gonadotropin combination is usually preferred when impaired clinical features are evident at baseline. This combined approach can reduce these differences, reaching similar ART outcomes. The results have been analyzed comparing both COS approaches and POSEIDN stratification. This latter shows clearly differences among POSEIDON groups.

## Data Availability

The datasets generated for this study are available on request to the corresponding author.

## Ethics Statement

The study was approved by the Independent Ethical Committee of the Humanitas Institutional Clinic (Milan, Italy). Consent was obtained from each patient after full explanation of the purpose and nature of all procedures used.

## Author Contributions

PL-S designed the project, collected data, and drafted the manuscript. IZ, AB, EZ, LS, AS, EM, RD, and AD collected clinical data. DS analyzed clinical data and drafted the manuscript. All authors participated to final manuscript.

### Conflict of Interest Statement

The authors declare that the research was conducted in the absence of any commercial or financial relationships that could be construed as a potential conflict of interest.
